# Calreticulin nuclear translocalization alleviates CaM/CaMKII/CREB signaling pathway to enhance chemosensitivity in HDAC inhibitor-resistant hepatocellular carcinoma cells

**DOI:** 10.18632/aging.204131

**Published:** 2022-06-20

**Authors:** Yi-Sheng Liu, Yu-Chun Chang, Wei-Wen Kuo, Ming-Cheng Chen, Tso-Fu Wang, Tung-Sheng Chen, Yueh-Min Lin, Chi-Cheng Li, Po-Hsiang Liao, Chih-Yang Huang

**Affiliations:** 1 Division of Hematology and Oncology, Department of Medicine, Kaohsiung Armed Forces General Hospital, Kaohsiung 802, Taiwan; 2 School of Medicine, National Defense Medical Center, Taipei 114, Taiwan; 3 Cardiovascular and Mitochondrial Related Disease Research Center, Hualien Tzu Chi Hospital, Buddhist Tzu Chi Medical Foundation, Hualien 970, Taiwan; 4 Department of Biological Science and Technology, College of Life Sciences, China Medical University, Taichung 406, Taiwan; 5 Ph.D. Program for Biotechnology Industry, China Medical University, Taichung 406, Taiwan; 6 Department of Surgery, Division of Colorectal Surgery, Taichung Veterans General Hospital, Taichung 407, Taiwan; 7 Faculty of Medicine, National Yang-Ming University, Taipei 112, Taiwan; 8 Department of Hematology and Oncology, Hualien Tzu Chi Hospital, Buddhist Tzu Chi Medical Foundation, School of Medicine Tzu Chi University, Hualien 97004, Taiwan; 9 School of Life Science, National Taiwan Normal University, Taipei 116, Taiwan; 10 School of Medicine, Chung Shan Medical University, Taichung 402, Taiwan; 11 Department of Surgical Pathology, Changhua Christian Hospital, Changhua 500, Taiwan; 12 Center of Stem Cell and Precision Medicine, Hualien Tzu Chi Hospital, Buddhist Tzu Chi Medical Foundation, Hualien 970, Taiwan; 13 Division of General Surgery, Department of Surgery, Shuang Ho Hospital, Taipei Medical University, New Taipei City 235, Taiwan; 14 Department of Medical Research, China Medical University Hospital, China Medical University, Taichung 404, Taiwan; 15 Graduate Institute of Biomedical Sciences, China Medical University, Taichung 404, Taiwan; 16 Department of Medical Laboratory Science and Biotechnology, Asia University, Taichung 413, Taiwan; 17 Center of General Education, Buddhist Tzu Chi Medical Foundation, Tzu Chi University of Science and Technology, Hualien 970, Taiwan

**Keywords:** calreticulin, hepatocellular carcinoma, HDACis-resistant cells, chemosensitivity

## Abstract

Calreticulin (CRT) is located in the endoplasmic reticulum (ER), it helps proteins fold correctly inside the ER, and acts as a modulator of Ca^2+^ homeostasis. Aberrant expression of CRT is implicated in several cancer types, qualifying CRT as a potential therapeutic target. However, it remains unclear how CRT affects specific oncogenic pathways. In this study, we used histone deacetylase inhibitors (HDACis) to establish drug-resistant liver cancer cells and further analyzed the molecular mechanism of development of drug resistance in those cells. The 2D gel electrophoresis and RT-PCR data showed that CRT was downregulated in HDACis-resistant cells by comparing with HA22T parental cells. We previously elucidated the development of drug-resistance in HCC cells via activation of PP1-eIF2α pathway, but not via ER stress pathway. Here, we show that thapsigargin induced ER stress through mechanism other than ER stress downstream protein GRP78-PERK to regulate CRT expression in HDACis-R cells. Moreover, the expression level of CRT was not the main cause of apoptosis in HDACis-resistant cells. Mechanistic studies identified the apoptosis factors in the nucleus—the HDACis-mediated overexpression of CRT, CRT translocation to the cell nucleus, and reduced CaM/CaMKII/CREB pathway—that led to chemosensitivity in HDACis-R HCC cells.

## INTRODUCTION

Several chemotherapeutic drugs are developed and approved by the Food and Drug Administration (FDA) in recent years against liver cancer, such as cabozantinib-s-malate [1], ramucirumab [2], pembrolizumab [3], lenvatinib mesylate [3], sorafenib tosylate [4], nivolumab [5], regorafenib [6], etc. Despite advances chemotherapeutic agents for treatment of liver cancer, development of drug resistance has remained the major impediment in the treatment of patients.

Most of the chemotherapeutic drugs approved for the treatment of malignant liver tumors inhibit specific tyrosine kinases, such as vascular endothelial growth factor receptors (VEGFR) [4, 7], Raf kinase [4, 7], platelet-derived growth factor (PDGF) [7], and PD-1 receptor [6]. However, epigenetic mechanisms are found to play a key role in cancer development [8]. Acetylation of lysine residues is one of the epigenetic histone modification events. Histone acetyltransferase (HATs)-mediated histone acetylation leads to chromatin decondensation and binding of transcriptional machinery to the DNA [9]. In contrast, histone deacetylation by histone deacetylases (HDACs) favors a more condensed, transcriptionally inactive chromatin structure [9]. These alterations affect both gene transcription and DNA repair. Thus, the development of epigenetic therapeutics is a potential and attractive domain in HCC.

In our previous studies, we used HDAC inhibitors (HDACis) to treat liver cancer cell. Moreover, we generated HDACis-resistant cells (HDACis-R cells) by treating HA22T cell line with apicidin, a novel HDAC-inhibitor, and investigated the molecular mechanisms of developing resistance in liver cancer cells [10–12]. The expression of phosphatase and tensin homolog (PTEN) [11], a tumor-suppressor gene, was decreased, while that of members of phosphoinositide 3-kinase (PI3K)-Akt pathway (a pro-survival protein) [10, 11] and EMT-like signaling (as a marker of metastatic) [12] was increased in HDACis-R cells than that in HDAC-sensitive cells. Therefore, these studies showed that HDACis act as potential therapeutic agents in HCC and elucidated the development of resistance.

Here, we aimed to further investigate the expression of calreticulin (CRT) that was differential between HA22T and HDACis-R cells [13]. CRT is a Ca^2+^-binding chaperone that helps proteins fold correctly in the lumen of endoplasmic reticulum (ER) and also functions as a multi-functional protein in non-ER activities, such as calcium homeostasis [14], cell adhesion [15], cell migration [16], transcriptional activities [17], and resistance to anoikis [18]. Several studies suggest CRT to be involved in the development of different cancer types via tumor generation and progression. For example, CRT affects cancer metastasis by interacting with integrins [19, 20], regulating cell proliferation via upregulation of proangiogenic VEGF in various cancer cells [21–23], and suppressing cell proliferation and enhancing cell differentiation in neuroblastoma cells [24]. However, CRT expressed (ecto-CRT) on the cell surface serves as a phagocytic signal for immunogenic cell death (ICD) mediated by dendritic cells (DCs) and cytotoxic T-cell activation [25–27].

Moreover, cytosolic CRT functions as a receptor for nuclear export of glucocorticoid receptor [28, 29] to suppress transcriptional activity of steroid hormone receptors in the development of hormone-dependent tumors [30]. However, how cytosolic CRT plays a role in the development of drug-resistant cancer remains unclear.

Calmodulin (CaM) is an intermediate for Ca^2+^-stimulated signaling cascades in many cell types. The Ca^2+^/CaM complex may activate a variety of enzymes and CaM Kinase (CaMK) family that control many cancer-related functions in several tumor types. Additionally, the CaMKII is a cancer marker that is frequently overexpressed and regulates cell proliferation, survival, invasion, and migration in various cancer types [31]. Our previous study demonstrated critical roles of CaM/CaMKII-γ pathway in the apicidin-persistent HCC prosurvival capability via ERK1/2/CREB/c-fos signaling activation [32]. These findings suggest that CaMKII-CREB is linked with differentiation, proliferation, apoptosis, and tumorigenicity; however, its role in the regulation of CRT remains unclear. Thus, we hypothesized that CRT may control drug-resistance in cancer cells by regulating the ER stress pathway or stimulating CRT translocation pathway. In the present study, CRT was overexpressed, which led to its translocation to the nucleus causing apoptosis in HCC cells treated with HDACis. The data showed that CRT was not regulated via ER stress pathway in HDACis-resistant HCC cells. Moreover, translocation of CRT, rather than its accumulation, led to apoptosis in HCC cells. These results indicate that CRT may serve as a target for developing anticancer therapeutics and preventive strategies by stimulating CRT translocation to the nucleus.

## RESULTS

### Analysis of the drug sensitivity and differential protein expression between parental and resistance HCC cells

**Figure 1:**
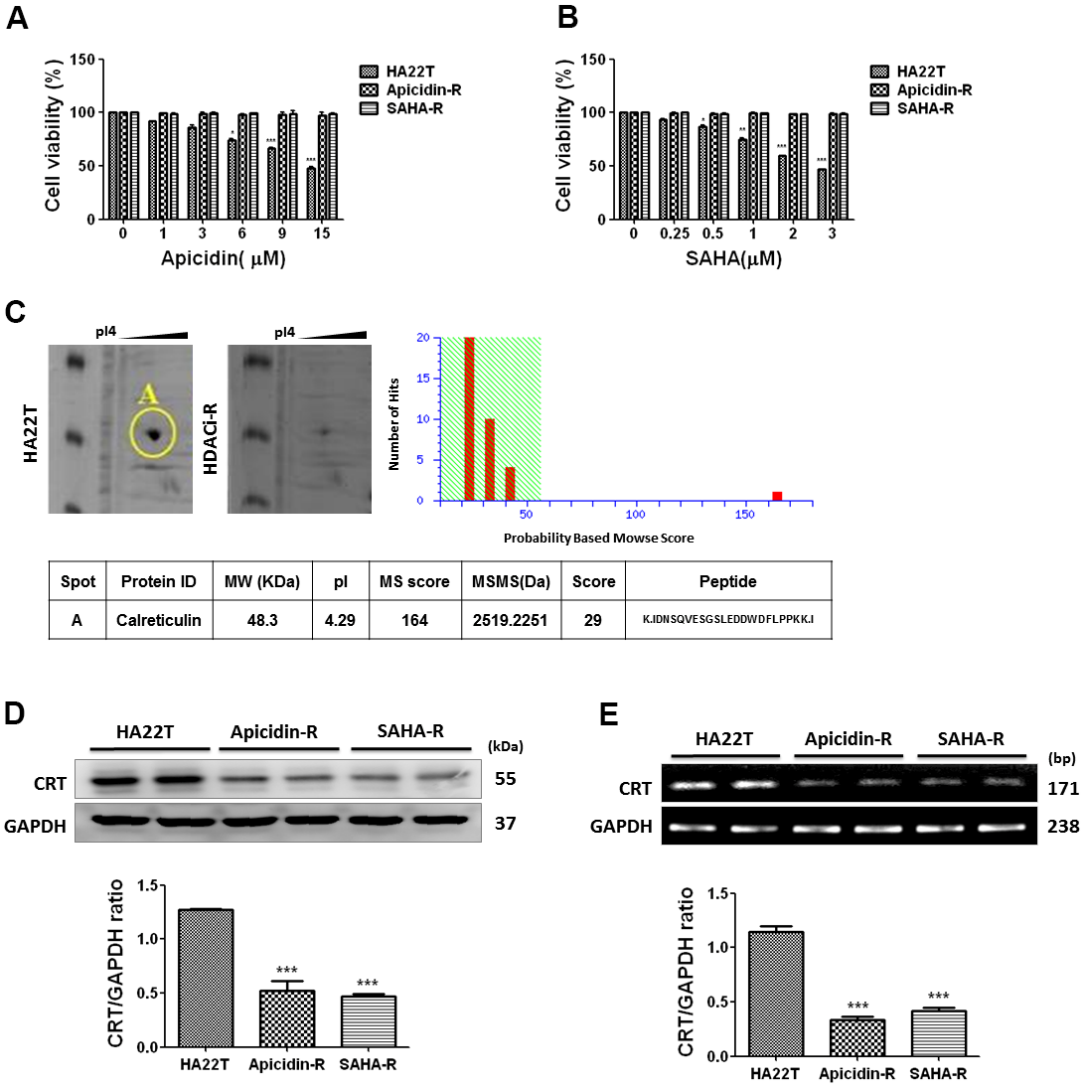
**HCC cells resistance to HDACis correlated with downregulation of CRT.** (**A**) Liver cancer cells are incubated with apicidin at various concentrations for 48 h and proliferation measured by MTT assay. (**B**) Liver cancer cells are incubated with SAHA at various concentrations for 48 h and proliferation measured by MTT assay. Data are expressed as percentage of the control and presented as mean ± S.D. of three independent experiments. (**C**) Two-dimensional gel electrophoresis analysis of proteins extracted from liver cancer cells, and further identification of candidate protein, calreticulin (CRT), by tandem mass spectrometer. (**D**, **E**) Western blotting and reverse transcription PCR analysis indicate decreased expression of CRT in HDACis-R cells than that in HA22T parental cells at protein and mRNA levels. Protein and mRNA expression are normalized to expression of GAPDH. *p <0.05, **p <0.01, ***p <0.001 compared with parental cell group.

First, we assessed the effect of HDACis on cell viability of HA22T and HDACis-R (apicidin-R and SAHA-R) cells using 3-(4, 5-dimethylthiazol-2-yl)-2, 5-diphenyltetrazolium-bromide (MTT) assay. Different concentrations of HDACis (apicidin and SAHA) were used to evaluate cell viability after incubating for 48 h. The analysis indicated that HDACis failed to reduce cell proliferation in HDACis-R cells; however, HDACis significantly attenuated the proliferation of HA22T parental cells in a dose-dependent manner (Figure 1A, 1B).

Following, we used two-dimensional gel electrophoresis (2-DE) to identify differentially expressed proteins, and selected a protein spot differential between HA22T and HDACis-R cells. The protein spot was analyzed by mass spectrometry and identified as calreticulin; it was significantly downregulated in the HDACis-R cells than in HA22T parental cells (Figure 1C). Furthermore, western blotting and qPCR analysis confirmed the downregulation of CRT in HDACis-R cells than in HA22T parental cells at protein and mRNA levels, respectively (Figure 1D, 1E). Therefore, we hypothesized that resistance to HDACis in HA22T cells correlated with downregulation of CRT.

### Overexpression of CRT enhances chemosensitivity to HDACis-mediated HCC cytotoxicity

**Figure 2:**
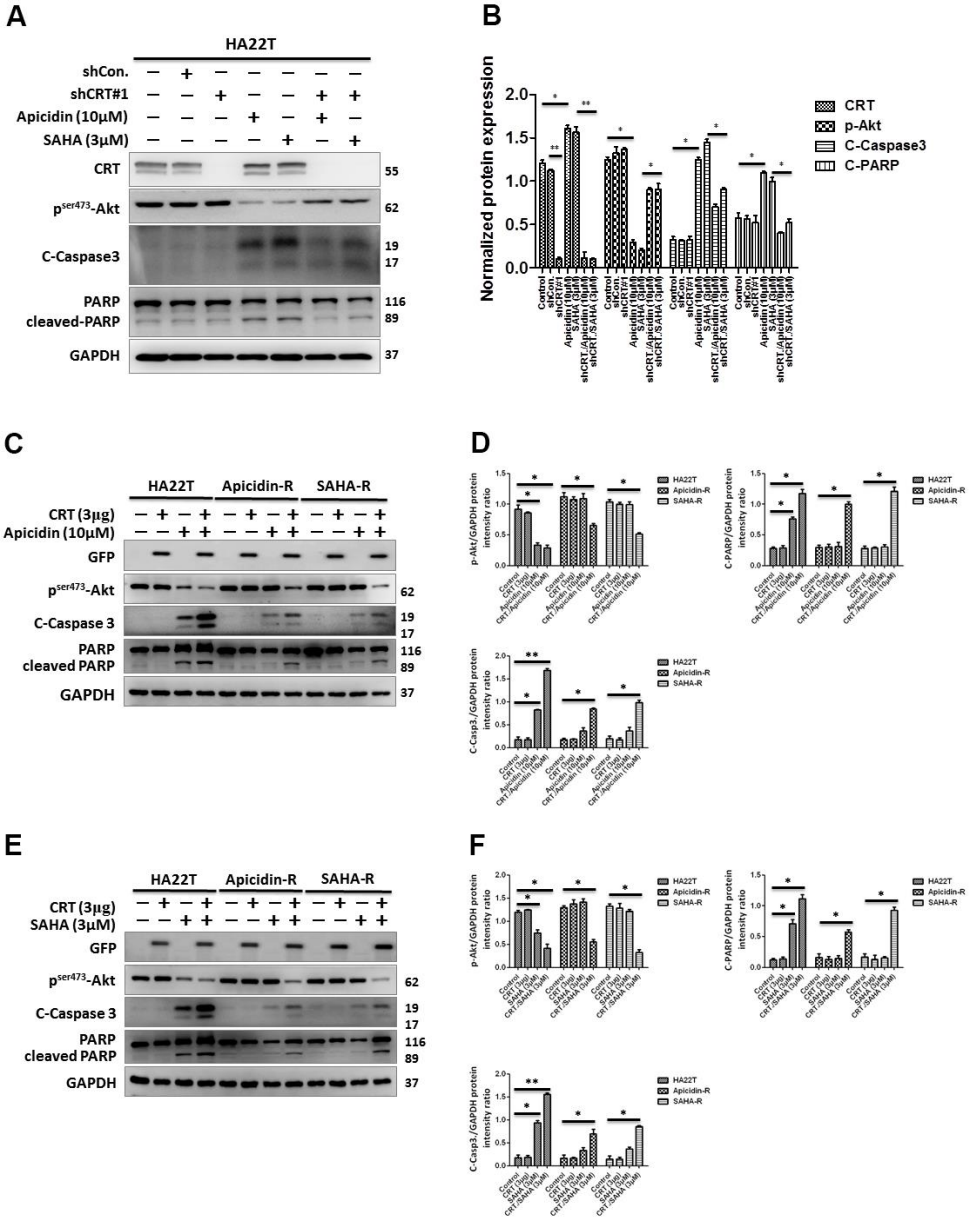
**Effect of overexpression and downregulation of CRT overexpression on cell apoptosis in HCC cells treated with HDAC inhibitors.** (**A**, **B**) shRNA-mediated knockdown of CRT enhances drug-resistance in HDAC inhibitor-treated cells than in HA22T parental cells. (**C**–**F**) Overexpression of CRT overexpression enhances HDAC inhibitor-induced chemosensitivity in HDAC inhibitor-resistant cells than in parental cells. All protein samples are analyzed using western blotting. Protein expression is normalized to expression of GAPDH. *p <0.05, **p <0.01, ***p<0.001 compared with control group.

Recent studies have shown that CRT plays a crucial role during tumor development. Therefore, we hypothesized that HDACis-R in HA22T cells was developed either by upregulation or downregulation of CRT. We performed short hairpin RNA (shRNA)-mediated knockdown and plasmid-based overexpression of CRT to examine its role in HA22T and HDACis-R cells. The analysis showed that knockdown of CRT had no significant effect on HA22T cells. The HDACis suppressed p-Akt levels, but the expression of cleaved caspase 3, cleaved PARP, and CRT increased in HA22T cells. Moreover, knockdown of CRT rescued HDACis-induced apoptosis by reducing levels of cleaved caspase 3 and cleaved PARP, and increased p-Akt levels in HA22T cells (Figure 2A, 2B). In contrast, transient transfection with CRT-plasmid showed no effect on the levels of p-Akt, cleaved caspase 3, and cleaved PARP. However, overexpression of CRT enhanced HDACis-induced apoptosis between HA22T and HDACis-R cells via decreased levels of p-Akt, along with increased expression of cleaved caspase 3 or cleaved PARP (Figure 2C–2F). Additionally, studies have demonstrated that CRT could be modified [33], degraded [34], and translocated to the nucleus [35]. Therefore, we investigated that whether the posttranslational modifications in CRT could enhance chemosensitivity in HCC cells. First, we assessed the effect of MG132 on the viability of HA22T and HDACis-R cells using MTT assay. The analysis indicated that MG132 (10μM) significantly attenuated viability of liver cancer cells in 24–48 h (Supplementary Figure 1A). We treated liver cancer cells with MG132 (10μM) for 8 h that induced the accumulation of CRT and posttranslational modifications in CRT via polyubiquitination between HA22T and HDACis-R cells (Supplementary Figure 1B, 1C). Moreover, CRT ubiquitination increased in the HDACis-R cells than that in HA22T parental cells after treatment with MG132 (10 μM) for 8 h and subsequent immunoprecipitation (IP)/immunoblotting (IB) analysis (Supplementary Figure 1B, 1C). Nevertheless, after incubation with HDACis (10 μM of Apicidin or 3 μM SAHA) for 24 h and addition of MG132 at the 16th hour enhanced chemosensitivity in HA22T parental cells, especially in the HDACis-R cells (Supplementary Figure 1D, 1E). The results indicate that stress stimulates translocation of CRT that is upregulated and further induces cell apoptosis.

### Expression of CRT is not regulated by ER stress pathway in HDACis-R cells

**Figure 3:**
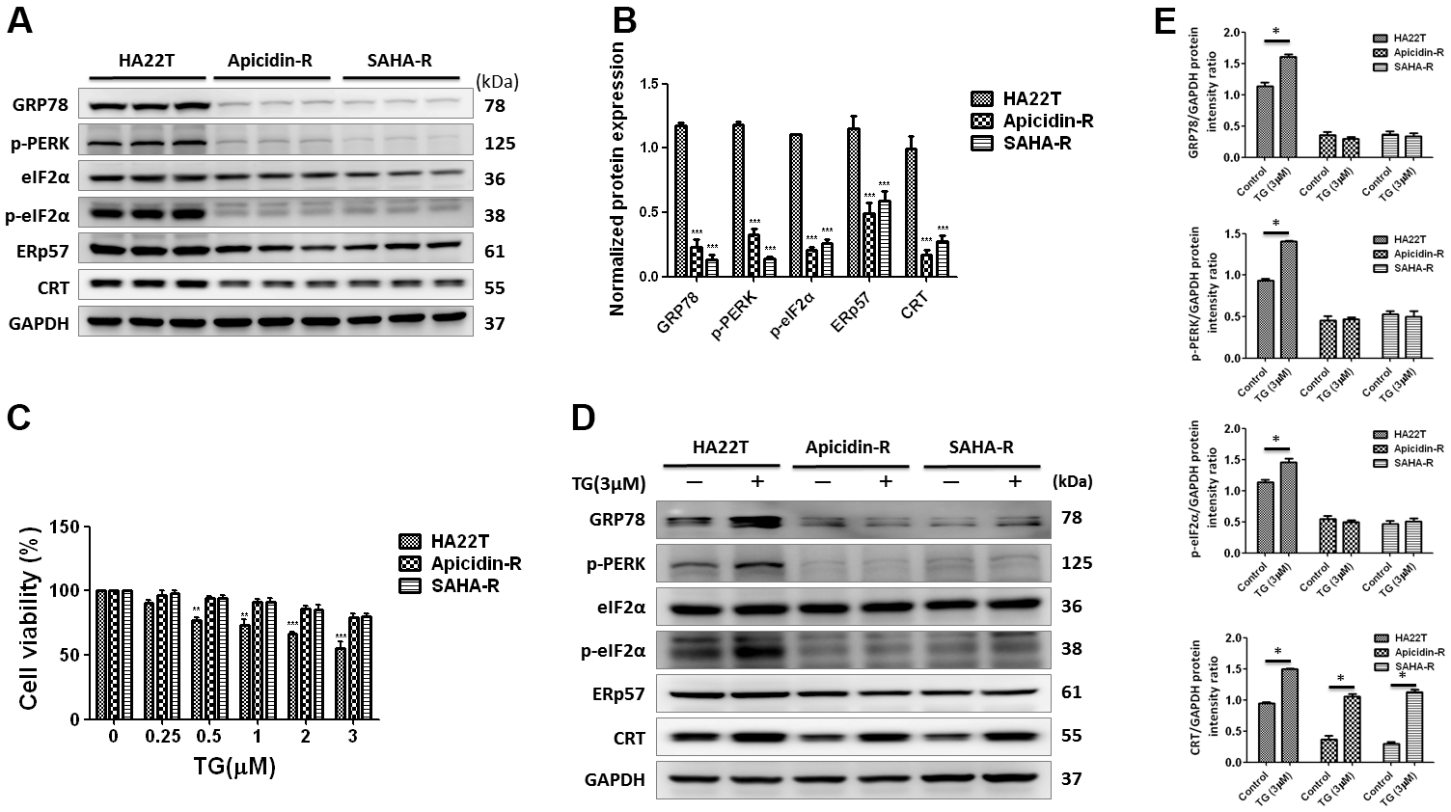
**ER stress-dependent pathway was not significantly involved in HDAC inhibitors-resistant HA22T cells.** (**A**, **B**) Protein expression levels of CRT-regulated ER stress pathway proteins (GRP78, p-PERK, p-eIF2, ERp57 and CRT) are measured by western blotting analysis. (**C**) Effect of thapsigargin (TG)-induced ER stress on the cell viability of liver cancer cells. Liver cancer cells are incubated with different concentration of TG for 24 h and cell viability is tested by MTT assay. (**D**, **E**) Expression of CRT-regulated ER stress pathway proteins (GRP78, p-PERK, p-eIF2, ERp57 and CRT) are measured using western blotting. All protein samples are analyzed by western blotting. Protein expression is normalized to expression of GAPDH. *p<0.05, **p<0.01, ***p<0.001 compared with control group.

Calreticulin resides mainly in the endoplasmic reticulum (ER), where it functions as a chaperone [36] and calcium homeostasis regulator [37]. To explore the molecular mechanism by CRT, we examined the protein expression level of the traditional ER stress pathways. The analysis indicated that expression of the ER stress signaling proteins (GRP78, p-PERK, p-eIF2, ERp57, and CRT) in the HDACis-R cells was downregulated than that in parental cells under normal conditions (Figure 3A, 3B). This indicated that the CRT expression was regulated by the ER stress pathway in HDACis-R cells.

To explore the regulation of CRT expression by the ER stress pathway activity, we treated the HCC cells with ER stress activator, thapsigargin (TG) [38] for 24 h. The analysis showed that TG could significantly attenuate HA22T parental cell viability in a dose-dependent manner, but not in apicidin-R cells (Figure 3C). TG initiated apoptosis via activation of p-PERK, a protein functioning upstream to the ER stress in HA22T parental cells. Moreover, expression of GRP78, p-PERK, and p-eIF2α was upregulated in HA22T parental cells than that in HDACis-R (Figure 3D, 3E), consistent with our previous observations with fisetin-induced chemosensitivity in HCC cells [39].

Additionally, expression of CRT was upregulated by TG between HA22T and HDACis-R cells. These results suggest that expression of CRT was not regulated via activation of the ER stress pathway—PERK-eIF2α—in HDACis-R cells.

### Nuclear translocation of calreticulin increases cell apoptosis in HDACis-R cells

To demonstrate that whether HDACis led translocation of CRT to the nucleus and further induced HCC apoptosis, we treated HCC cells with HDACis (10 μM of Apicidin or 3 μM of SAHA) and measured protein levels of the members of CaM/CaMKII/CREB signaling pathway in both the cytosolic and nuclear fractions. The results showed that HDACis significantly induced translocation of CRT to the nucleus and further led to apoptosis via attenuation of the CaM/CaMKII/CREB signaling pathway in the nuclear fraction of HA22T parental cells, but not in the HDACis-R cells (Figure 4A, 4B). Next, we transiently transfected a CRT plasmid (3 μg) in HCC cells for 24 h before treating them with HDACis to investigate whether overexpression of CRT could enhance HDACis-induced chemosensitivity in HDACis-R cells. The analysis showed that overexpression of CRT was not significantly affected between HA22T and HDACis-R cells. Moreover, HDACis significantly induced apoptosis by initiating translocation of CRT to the nucleus and further attenuating CaM/CaMKII/CREB signaling pathway between CRT-transfection in HA22T and HDACis-R cells in nuclear fractions (Figure 4C, 4D). Further, using TUNEL assay, we found that HDACis only induced cell apoptosis in HA22T cells than in control cells. Overexpression of CRT and treatment with HDACis significantly increased the TUNEL-positive cells than with HDACis group between HA22T and HDACis-R cells (Figure 4E–4H). Additionally, abnormal calcium signaling is related to occurrence of various cancer types and therapy resistance [40, 41]. However, it remains unclear how CRT affects calcium regulation in the nucleus. To determine whether CRT overexpression affected nuclear calcium levels in HDACi-mediated HCC cytotoxicity, we measured calcium levels in the HCC cells by using calcium detection reagents. The analysis demonstrated that HDACis significantly induced calcium accumulation in HA22T parental cells. Moreover, HDACis significantly enhanced translocation of CRT to the nucleus and led to increased calcium accumulation in CRT-transfected HCC cells than that in untransfected cells (Figure 4I–4K). Taken together, these findings indicate that HDACis induce translocation of CRT to the nucleus and further caused cell apoptosis via CaM/CaMKII/CREB signaling activation, and also enhance nuclear calcium accumulation in CRT-transfected HCC cells.

**Figure 4:**
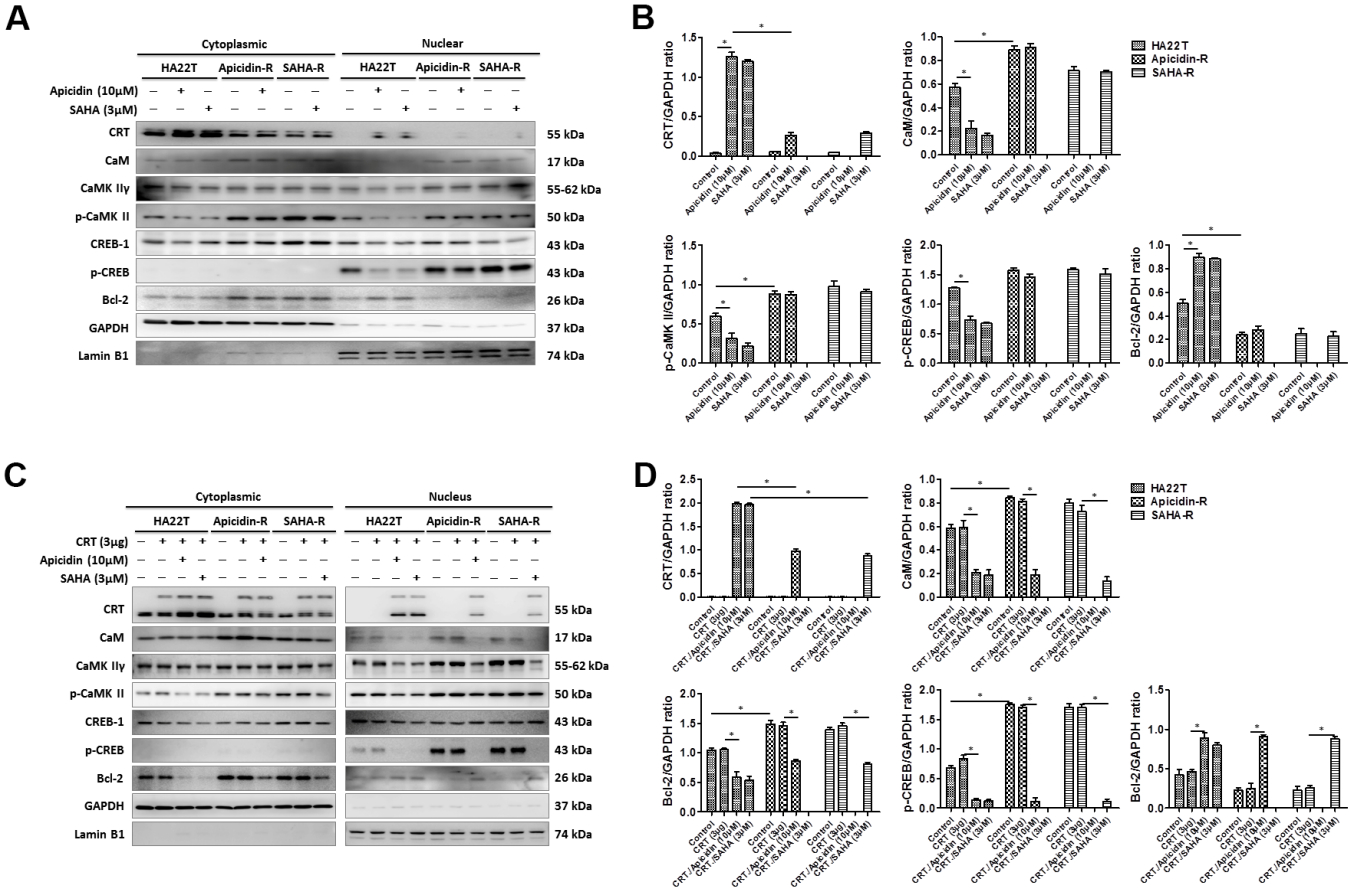
**HDAC inhibitors trigger CRT translocation to the nucleus in liver cancer cells overexpressing CRT.** (**A**, **B**) HDAC inhibitors activate CRT nuclear translocation and inhibit CaM/CaMKII/CREB signaling pathway in the nuclear isolate of the HA22T parental cells. (**C**, **D**) HDAC inhibitors activate CRT nuclear translocation and inhibit CaM/CaMKII/CREB signaling pathway in the nuclear isolate of HCC cells overexpressing CRT.

**Figure 4:**
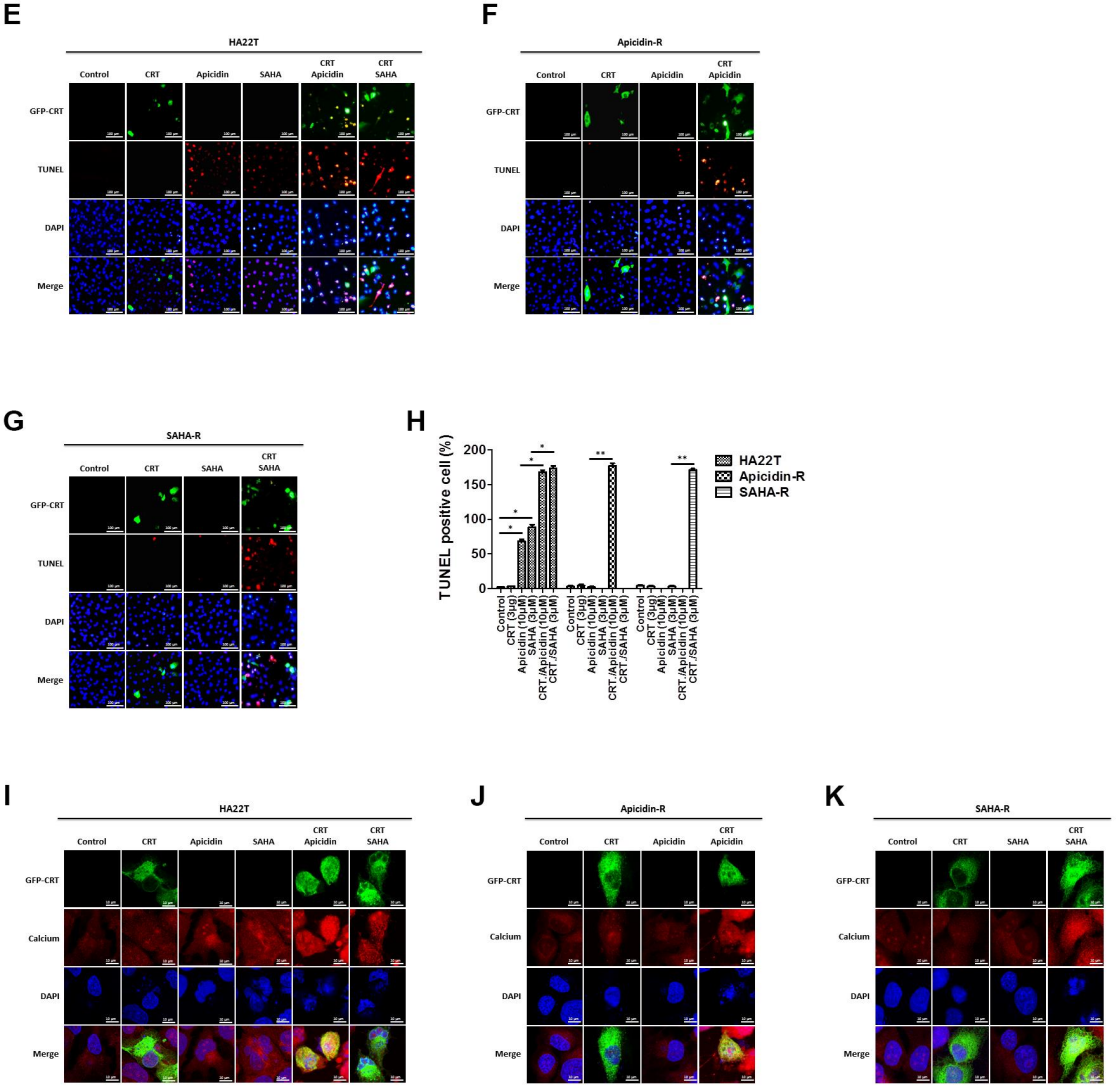
**HDAC inhibitors trigger CRT translocation to the nucleus in liver cancer cells overexpressing CRT.** (**E**–**G**) HCC cells treated with vehicle, transfected with CRT-plasmid, or HDAC inhibitors alone or in combination exhibit DNA damage, as determined by TUNEL assays. Scale bar = 100 μm. Upper panel: Green fluorescence indicates CRT-transfected HCC cells. HCC cells are stained with fluorescein-labeled dUTP according to the protocols reported in the Methods section. Red fluorescence indicates TUNEL-positive cells. Lower panel: HCC cells in the upper panel are stained with 4',6-diamidino-2-phenylindole (DAPI) to identify cell nuclei. (**H**) Rate of apoptosis is calculated as the percentage of TUNEL-positive cells among the total number of counted parental and resistant cells (mean ± SD, n = 3). (**I**–**K**) Cells are seeded in an 8-well chamber and treated with vehicle, CRT-plasmid, or HDAC inhibitors alone or in combination. HDAC inhibitors trigger CRT translocation to the nucleus and stimulate calcium accumulation, analyzed by confocal microscopy. Scale bar = 10 μm. Mean values are significantly different than that in control group. *p< 0.05, **p< 0.01, ***p< 0.001.

## DISCUSSION

In this study, we selected CRT was found to be a differentially expressed protein between the HA22T parental cells and HDACis-resistant cells previous studies. Many studies showed CRT to be highly expressed in several cancer types [42], such as high level microsatellite instability colorectal adenocarcinomas [43], esophageal squamous cell carcinoma [16], oral squamous cell carcinoma [44], breast cancer [45], and acute myeloid leukemia [46]. In contrast, some studies observed downregulation of CRT in laryngeal squamous cell carcinoma lesions [47], endometrial cancer [48], vaginal and cervical carcinoma [49], and non-small-cell lung cancer [50]; expression of CRT is especially low in advanced human prostate cancer and ovarian serous carcinoma than that in benign tissue [51, 52]. Therefore, expression of CRT may correlate with carcinogenesis and cancer progression. The present study showed the expression of CRT to be lower in HDACis-R cells than that in HA22T parental cells at the protein and mRNA levels, suggesting it to be a new therapeutic target in HCC. The function of CRT is to bind to URP and induces ER stress in the UPR pathway via IRE-1α, XBP-1 and ATF-6. We detected the IRE-1, XBP-1 and ATF-6 expression after treated with thapsigargin in HCC cells. The results indicated that expression of IRE-1, XBP-1 and CHOP were higher in HA22T cells than in HDACi-R cells (Supplementary Figure 2A, 2B). Additionally, IRE-1 and CHOP expression upregulated after treated with TG in HCC cells but XBP-1 and ATF-6 were not. These results suggest that CHOP-induced apoptosis in HDACis-R cells were not mediated through PERK, XBP-1, ATF-6 signaling pathway.

Overexpression of CRT can significantly suppress cell viability and proliferation in MCF-7 [53] and neuroblastoma cells [54]. Here, overexpression of CRT-alone showed no effect on cell apoptosis. Interestingly, overexpression of CRT enhanced chemosensitivity in HDACis-mediated HCC cytotoxicity. Furthermore, CRT can be localized to the plasma membrane and nucleus either as native or post-translationally modified isoforms, performing diverse and important non-ER functions [55]. For instance, CRT is counterbalanced by CD47 for the adaptive immune response on the cell surface [56]. We also explored the role of UB-CRT in HCC apoptosis. The studies indicated that the levels of posttranslational modifications in CRT were higher in HDACi-R cells; also UB-CRT may lead to increased apoptosis in HDACis-mediated HCC cells; but UB-CRT-alone failed to affect the expression level of these proteins. CRT was stabilized by integrin-ligand binding on the extracellular matrix (ECM) and activated focal adhesion kinase (FAK) for regulating cell-adhesion [57, 58]. To understand how CRT regulates cell apoptosis, we evaluated the effect of CRT expression on ER stress pathway. Recent studies found that CRT is an ER-binding protein regulated by ERP57 [59–62]. Here, we observed that in HDACis-R cells, the expression of GRP78, p-PERK, p-eIF2α, ERp57, and CRT was significantly lower than that in HA22T parental cells. Therefore, we used TG (one of the most popular inducers of ER stress) in HA22T parental cells and HDACis-resistant cells. TG is a specific inhibitor of the sarcoplasmic/endoplasmic reticulum Ca^2+^-ATPase (SERCA) that causes ER stress through depletion of Ca^2+^ store in the ER [63–66]. CRT acts as a chaperone and affects Ca^2+^ homeostasis [67], which influences gene expression and cell adhesion [68]. CRT activated the Wnt and CaM/CaMK II pathways by regulating Ser/Thr phosphatase in regulating cellular adhesion, growth, migration, and differentiation [69–71]. Previous studies indicated that decreased CRT expression levels were paralleled by an increase in CaM abundance, with concomitant decrease in cell spreading [69]. Notably, inhibition of CRT expression could activate c-src expression compared to the overexpressing cells. Activation of c-src could increasing the affinity of calmodulin for CaMK II [72, 73]. However, Tyrosine phosphorylated calmodulin effectively activates CaMK II and this tyrosine phosphorylation event is inhibited by high calcium concentration [74, 75]. Slight differences in Ca^2+^ signaling mechanisms can disrupt cell function, such as using too much/ little Ca^2+^ at the wrong time/place can lead to rapid cell death by necrosis, or inducing cell apoptosis. Following this suggestion, we check the expression of Ca channel-related markers in HCC cells and obtained that treated with HDAC inhibitors up-regulation of Ca channel-related markers in the HA22T cells but no effect in HDACis-R cells (Supplementary Figure 3A–3D). Interestingly, treated with HDAC inhibitors in overexpression of CRT HCC cells up-regulated expression of L-type Ca++ CP α1C, SERCA2 and IP3R in HCC cells. These results hint that HCC cells resistance to HDACi through CRT expression but not related with Ca channel. In the current study, overexpressed CRT translocated to the nucleus and activated the CaM/CaMKII/CREB pathway in HDACi-mediated HCC cytotoxicity. Additionally, calcium accumulation activated cell apoptosis signaling by CRT in the nucleus. The study findings can aid in clinical treatment: measurement of CRT levels and determination of sensitivity to HDACis before chemotherapy would help in better planning the therapeutic scheme for effective treatment of HCC.

**Figure 5:**
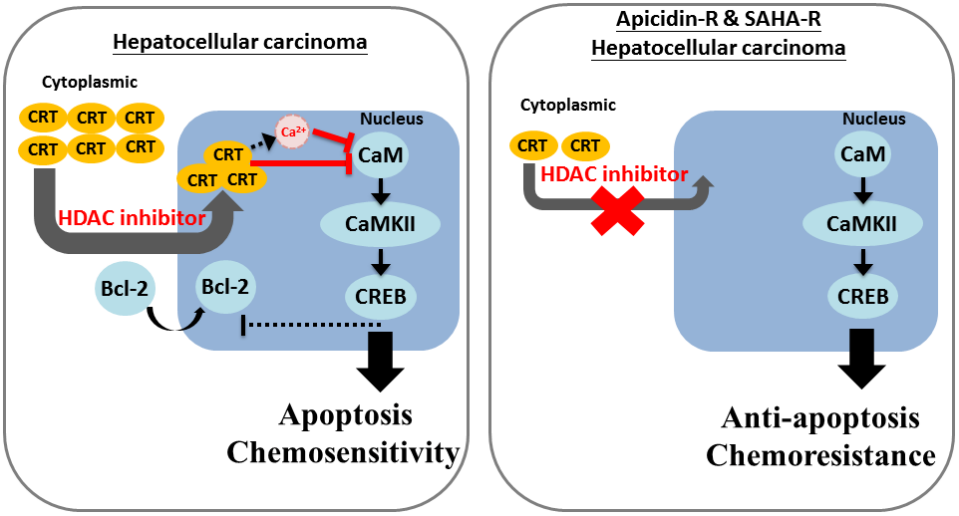
**Mechanism of CRT-mediated chemosensitivity in HCC cells via nucleus translocation and reduction in CaM/CaMKII/CREB signaling.** We revealed that decreased expression of CRT lead to down-regulated the chemosensitivity of HDACi in HCC cells. Moreover, we also obtain that CRT translocation into the nucleus is an important mechanism of chemoresistance and HDACi-induced cell apoptosis in HCC cells.

In conclusion, the study suggests that HDACis induce cell death by increasing cytoplasmic localization of CRT in the nucleus and further enhance calcium accumulation and CaM/CaMKII/CREB pathway suppression in HA22T parental cells. In contrast, low cytoplasmic levels of CRT prevent its nuclear translocation by HDACis, leading to chemoresistance and suppression of apoptosis in HDACis-R cells (Figure 5). Moreover, we identified a new drug resistance mechanism mediated by suppression of CRT translocation to the nucleus. In response to treatment with HDACis, reduced expression of CRT suppresses its translocation to the nucleus, inhibiting calcium accumulation and activation of CaM/CaMKII/CREB pathway. These results suggest that stimulating CRT translocation to the nucleus can be a novel target for treating drug resistance in liver cancer.

## MATERIALS AND METHODS

### Cell culture and generation of HDACis-resistant cells

HA22T (BCRC, Taiwan; Cat. No. 60168) or HDACis-resistant (HDACis-R; apicidin-R and SAHA-R) cells were cultured in Dulbecco’s Modified Eagle’s Medium (DMEM; Sigma, St. Louis, MO, USA, Cat. No. D5523) supplemented with 10% FBS (characterized fetal bovine serum, Millipore, Billerica, MA, USA) and 1% penicillin (Corning, NY, USA, Cat. No. 30-002-CI) in humidified air with 5% CO_2_ at 37° C. The HDACis-R cells were established from HA22T cells by following the protocol elaborated in our previous report [10, 11, 39, 76].

### Cell viability assay

Cells (1×10^4^/well) were plated in 24-well plates and treated with increasing concentrations of apicidin, SAHA and thapsigargin for 48 h, 48 h and 24 h, respectively. Furthermore, a 10 μM concentration of MG132 (carbobenzoxy-Leu-Leu-leucinal, Sigma-Aldrich Inc., St. Louis, MO, USA) was used to treat liver cancer cells at different time points. A 0.5 mg/mL concentration of yellow colored 3-(4, 5-dimethylthiazolyl-2)-2, 5-diphenyltetrazolium bromide (MTT; Sigma-Aldrich Inc., St. Louis, MO, USA) was added to each well for 3 h in humidified air with 5% CO_2_ at 37° C, until conversion into formazan crystals via mitochondrial dehydrogenase enzymes [77]. The purple MTT-formazan crystals were dissolved in 200 μL of DMSO and the formazan level was measured as absorbance at 570 nm using an automated microplate reader (Thermo Fisher Scientific Inc., Grand Island, NY, USA).

### Reverse transcription PCR

Total RNA was isolated and purified from cells using Quick-RNA™ MiniPrep kit (Zymo Research, Irvine, CA, USA), according to the manufacturer’s protocol. A total of 1 μg of RNA from each sample was reverse transcribed with a TaqMan™ reverse transcription kit (part no. N808-0234; Applied Biosystems), following the manufacturer’s protocol; and placed the PCR tubes on a thermal cycler (Thermo Fisher Scientific Inc., Grand Island, NY, USA) and incubated at 42° C for 65 min, 90° C for 5 min, and then at 4° C. The synthesized cDNAs were further amplified by RT-PCR using CRT primers (Forward: 5’-CGTCTACTTCAAGGAGCAGT-3’, Reverse: 5’-AGAGCATAAAAGCGTGCATC-3’, 171 bp), GAPDH primers (Forward: 5’-AAG GCTGTGGGCAAGG-3’, Reverse: 5’-TGGAGGAGTGGGTGTCG-3’, 238 bp), with the ProTaq System using ProTaq Master Mix (Protech Technology Enterprise CO., Ltd., Taiwan). One μL of the cDNA was amplified in a total volume of 25 μL reaction mixture containing 10× Reaction buffer (2.5 μL), 25 mM MgCl_2_ (1.5 μL), 10 mM dNTP mixture (0.5 μL), and 10 μM of forward and reverse primers. The RT-PCR cycles were estimated in a preliminary study and optimized in the PCR exponential phase. The conditions were as follows: initiation, 95° C for 5 min; followed by 28 cycles of denaturation at 95° C for 30 sec, annealing at 54° C for 30 sec, and extension at 72° C for 50 sec; final extension, 72° C for 5 min; stop reaction, 4° C. PCR product was analyzed by gel electrophoresis on a 1.5% agarose gel. Gene expression was normalized to expression of GAPDH in each sample.

### Two-dimensional (2D)-gel electrophoresis

Total protein was dissolved in urea lysis buffer containing 1% protease inhibitor cocktail, sonicated, and centrifuged at 17,000 ×*g* for 30 min. We used an 18-cm Immobiline DryStrip (GE Healthcare Life Sciences Inc., Pittsburgh, PA, USA) and IPGphor isoelectric focusing unit (GE Healthcare Life Sciences Inc.) at 62000 Vh to perform isoelectric focusing of protein (100 μg) obtained from gradient-purified total protein. A 12.5% sodium lauryl sulfate-polyacrylamide gel electrophoresis was used for second-dimension separation and used silver staining to detect the protein. Targeted protein spots were excised from the gel and subjected to in-gel digestion with trypsin to recover the peptide. The peptides were identified and analyzed by electrospray ionization mass spectrometry (MS/MS) from Proteomics Core Laboratory (Office of Research and Development, China Medical University, Taiwan).

### Antibodies and drug formulations

The following antibodies were used in the study: anti-GRP78 (sc-1050, Santa Cruz, CA, USA), anti-p-PERK Thr981 (sc-32577, Santa Cruz), anti-eIf2α (sc-133132, Santa Cruz), anti-CRT (sc-166837, Santa Cruz), anti-ERp57 (sc-28823, Santa Cruz), anti-Ub (sc-8017, Santa Cruz), anti-p-Akt1/2/3 (sc-7985, Santa Cruz), anti-GAPDH (sc-32233, Santa Cruz), anti-GFP (sc-8334, Santa Cruz), anti-Lamin B1 (sc-374015, Santa Cruz), anti-CaM (sc-137079, Santa Cruz), anti-CaMKIIγ (sc-1541, Santa Cruz), CREB-1 (sc-186, Santa Cruz), anti-p-CaMKII (ab182647, abcam, Waltham, MA, USA), anti-Bcl-2 (610539, BD Biosciences, Franklin Lakes, NJ, USA), anti-cleaved-caspase 3 (9664, Cell Signaling Technology, Danvers, MA, USA), anti-p-CREB Ser133 (9198, Cell Signaling Technology), anti-p-eIF2α Ser51 (9721, Cell Signaling Technology), and anti-PARP (9542, Cell Signaling Technology). All secondary antibodies (HRP-conjugated anti-rabbit, anti-mouse, and anti-goat) were purchased from Sigma (Sigma-Aldrich Inc., St. Louis, MO, USA). All the drug formulations were purchased from Sigma (Sigma-Aldrich Inc.).

### Western blotting analysis

Cell samples were extracted with lysis buffer (20 mM Tris base, 1% NP-40, 150 mM NaCl, 1 mM EDTA, 0.1% phosphatase inhibitor, and proteinase K inhibitor). Cell pellets were lysed for 30 min on ice and clarified by centrifugation at 12,000 rpm for 20 min, and protein concentration was measured using Bradford protein assay (Bio-Rad, Hercules, CA, USA). Further, an aliquot of each sample extraction equivalent to 20 μg of protein was boiled after adding an appropriate amount of 5-fold protein sample buffer (40 % glycerol, 10% SDS, 5% β-mercaptoethanol, 0.02% bromophenol blue, and 0.5 M Tris-HCl pH 6.8). The proteins were separated using 8–12% SDS-polyacrylamide gels, and transferred to PVDF membranes (Millipore, Belford, MA, USA) using Bio-Rad electrotransfer system (Bio-Rad Laboratories Inc., Hercules, CA, USA). The membranes were blocked with blocking buffer [78] (5 % non-fat milk, 20 mM Tris-Base, pH 7.4, 150 mM NaCl, and 0.1% Tween-20) at room temperature for 1–2 h and probed with specific primary antibodies at 4° C overnight. Furthermore, the protein bands were measured with HRP-conjugated secondary antibodies (Sigma-Aldrich, Inc.) at room temperature for 1 h and using custom-made ECL (Millipore, Billerica, MA, USA) detection reagents to detect the target protein on immunoblot PVDF membranes by the ImageQuant LAS 4000 digital imaging system (Digital Imaging System, Commerce, CA, USA).

### Immunoprecipitation assay

The same procedure, as described for western blotting, was used to obtain the total protein. Further, 50 μL of the Protein G beads slurry was transferred to an Eppendorf tube and washed three times with TBST buffer (20 mM Tris-Base, 150 mM NaCl, 0.1 % Tween-20, and pH 7.4). Next, 2 μg of primary antibody was added in 250–500 μL of TBST buffer and transferred to an Eppendorf tube containing the Protein G beads slurry and subjected it to IP rotor at 4° C for 6 h. Furthermore, the contents were washed three times with TBST buffer and >500 μg of protein sample was added in an Eppendorf tube containing the Protein G beads slurry and subjected the samples to IP rotor at 4° C overnight. Finally, the supernatant was completely and carefully removed and the beads were washed 3–5 times with TBST buffer, and 50 μL of 1-fold sample buffer was boiled at 100° C for 10 min, followed by western blotting to separate the proteins.

### Plasmid and gene transfection

Plasmid (pCMV3-CALR-GFPSpark) encoding calreticulin was purchased from Asia Bioscience Co., Ltd., Taiwan. An siRNA against CRT was purchased from Sigma. The cells were 60–70 % confluent at the time of transfection. The plasmid or siRNA of CRT were transfected in the cells using jetPRIME® (Polyplus Transfection Inc, Illkirch, France) transfection reagent, according to the manufacturer’s protocol. The cells were incubated in humidified air with 5 % CO_2_ at 37° C for 24 h.

### Cytoplasmic and nuclear extraction

Cells were grown in 100 mm dishes, and at 60–70 % confluent growth, were transfected with 3 μg of plasmid for 24 h, and treated with 10 μM of apicidin or 3 μM of SAHA for 24 h. Cytoplasmic and nuclear fractions were obtained using the Nuclear/Cytosol Fractionation Kit (BioVision, Milpitas, CA, USA), following the manufacturer’s protocol. The extracted proteins were stored at -80° C for further western blotting assay.

### TUNEL assay

Cells were grown in 8-well chamber slides (MatTek; Ashland, MA, USA) and transfected with pCMV3-CALR-GFPSpark (Asia Bioscience Co. Ltd. HG13539-ACG) for 24 h, and treated with HDACis (10 μM apicidin or 3 μM SAHA) for 24 h. We measured apoptosis/ DNA damage in HA22T cells or HDACis-resistant cells using *In Situ* Cell Death Detection Kit and TMR red (Roche, Basel, Switzerland), according to the manufacturer’s instructions [79]. Fluorescence was visualized using a fluorescence microscope coupled to an image analysis system. Four microscopic fields were randomly-selected, and average counts of TUNEL-positive cells were calculated.

### Intracellular calcium staining

Cells were seeded and transfected with pCMV3-CALR-GFPSpark (Asia Bioscience Co. Ltd. HG13539-ACG) for 24 h in chamber slides, and treated with HDACis (10 μM apicidin or 3 μM SAHA) for 24 h. Cells were stained with 5 μM of Cal-630™ AM kit (AAT Bioquest Inc., Sunnyvale, CA, USA), and fixed with 4 % paraformaldehyde for 30 min at room temperature. Finally, the samples were washed by PBS for three times and stained with Fluoroshield™ with DAPI (blue) (Sigma-Aldrich, Inc.) as nuclear counterstain. The samples were analyzed under a fluorescence microscope with an excitation wavelength of 475 nm (green) to detect CRT translocation, an excitation wavelength of 600/640 nm to detect calcium, and UV light microscope to detect cell nucleus (blue).

### Statistical analysis

Statistical analysis was performed by one-way analysis of variance (One-way ANOVA) and SigmaPlot 10.0 software (Systat Software Inc., San Jose, CA, USA), along with GraphPad Prism 8. In all tests, *p* values < 0.05 were considered as statistically significant. * *p* < 0.05, **p < 0.01, and ***p < 0.001.

## Supplementary Material

Supplementary Figures
