# Supplementary material for: Calreticulin nuclear translocalization alleviates CaM/CaMKII/CREB signaling pathway to enhance chemosensitivity in HDAC inhibitor-resistant hepatocellular carcinoma cells
Source: Aging (Albany NY). 2022 Jun 20;14(12):5097–115. doi: 10.18632/aging.204131 (PMC9271289; doi:10.18632/aging.204131)
Supplement: Supplementary Figures [file aging-14-204131-s001.pdf]

SUPPLEMENTARY FIGURES

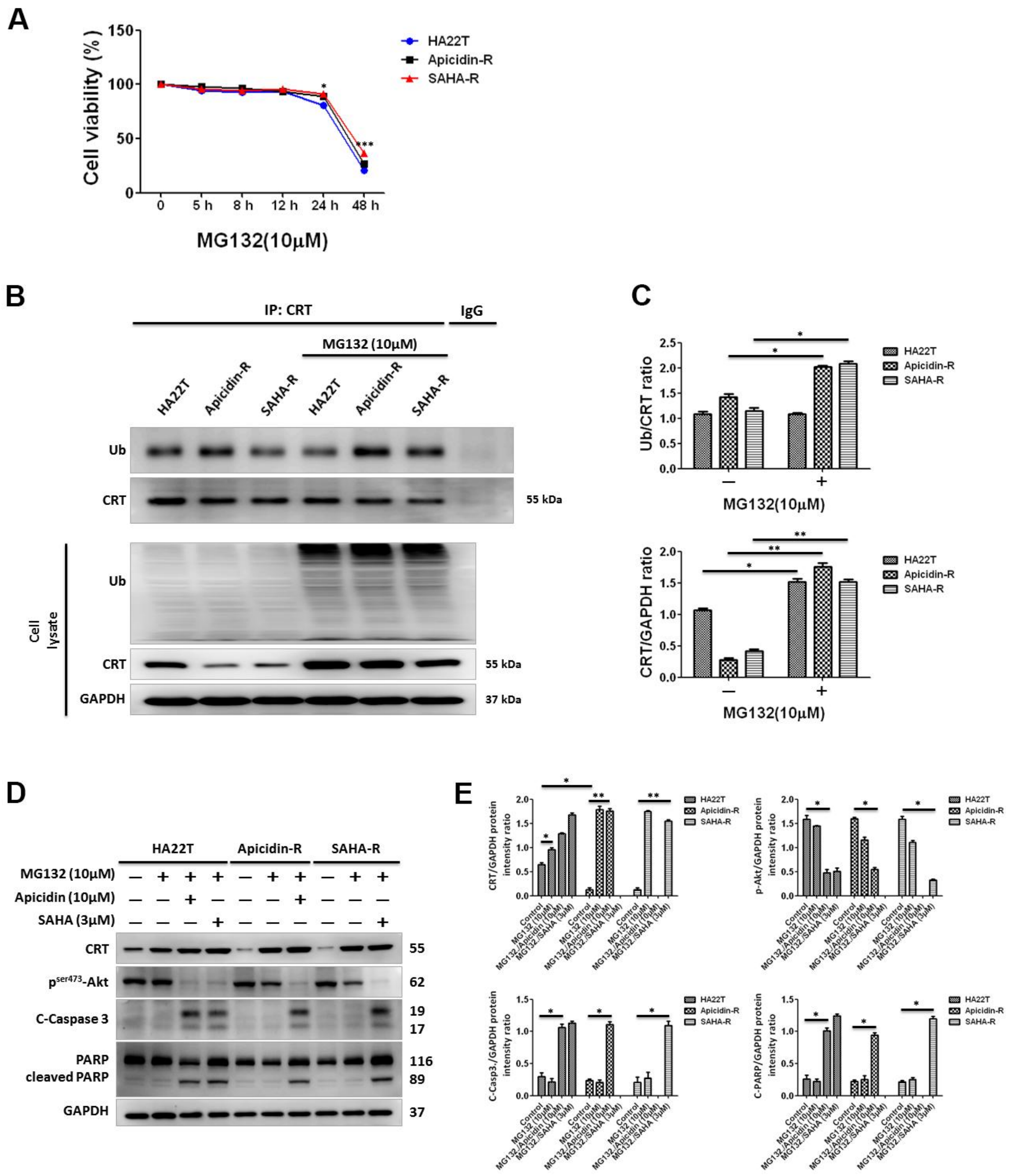

Supplementary Figure 1. Post-translational modifications of CRT enhance chemosensitivity in liver cancer cells after stimulation with HDACis. (**A**) Effect of MG132-induced apoptosis on cell viability of liver cancer cells. Cells are incubated with MG132 (10 µM) for different time points and cell viability is tested using MTT assay. (**B**, **C**) Liver cancer cells are treated with MG132 (10 µM) for 8 h. Cell lysates are prepared for immunoprecipitation (IP) with an anti-CRT antibody followed by immunoblotting (IB) using indicated antibodies. CRT is modified via the ubiquitination pathway in HDACis-resistance cells as observed in the IP/IB analysis. All protein samples are analyzed by western blotting. Protein expression is normalized to that of CRT in IP/IB analysis, and expression of the other proteins (including cell lysate) is normalized to that of GAPDH. *p<0.05, **p<0.01, ***p<0.001 compared with control group. (**D**, **E**) Liver cancer cells are treated with HDAC inhibitors (10 µM of apicidin or 3 µM SAHA) for 24 h and MG132 is added at the 16th hour. The expression of apoptotic proteins is assessed by western blotting. All protein samples are analyzed by western blotting. Protein expression is normalized to expression of GAPDH. *p<0.05, **p<0.01, ***p<0.001 compared with control group.

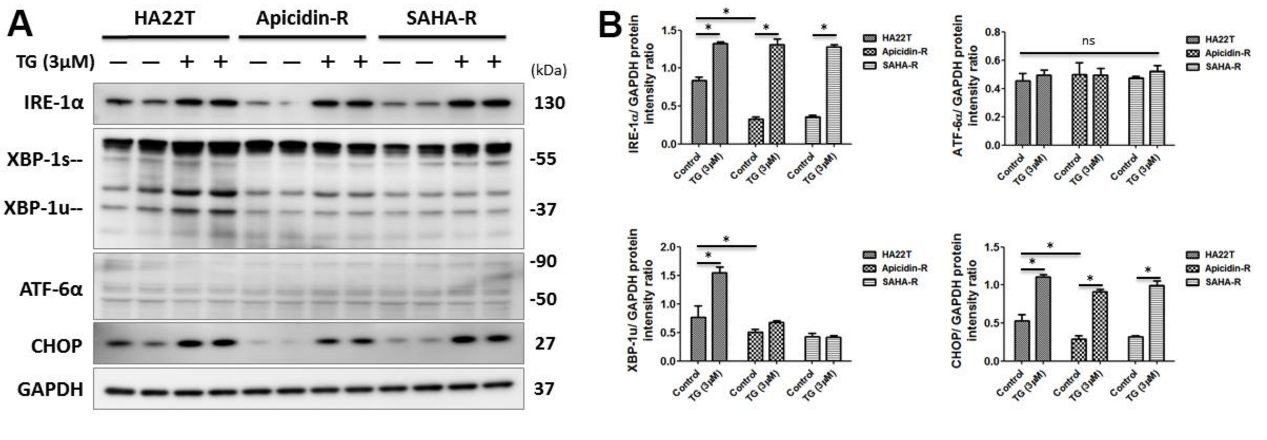

Supplementary Figure 2. The function of CRT is to bind to URP and induces ER stress in the UPR pathway via IRE-1/, XBP-1 and ATF-6. (**A**, **B**) Expression of CRT-regulated ER stress pathway proteins (IRE-1α, XBP-1, ATF-6 and CHOP) are measured using western blotting. All protein samples are analyzed by western blotting. Protein expression is normalized to expression of GAPDH. **p*<0.05, ***p*<0.01, ****p*<0.001 compared with control group.

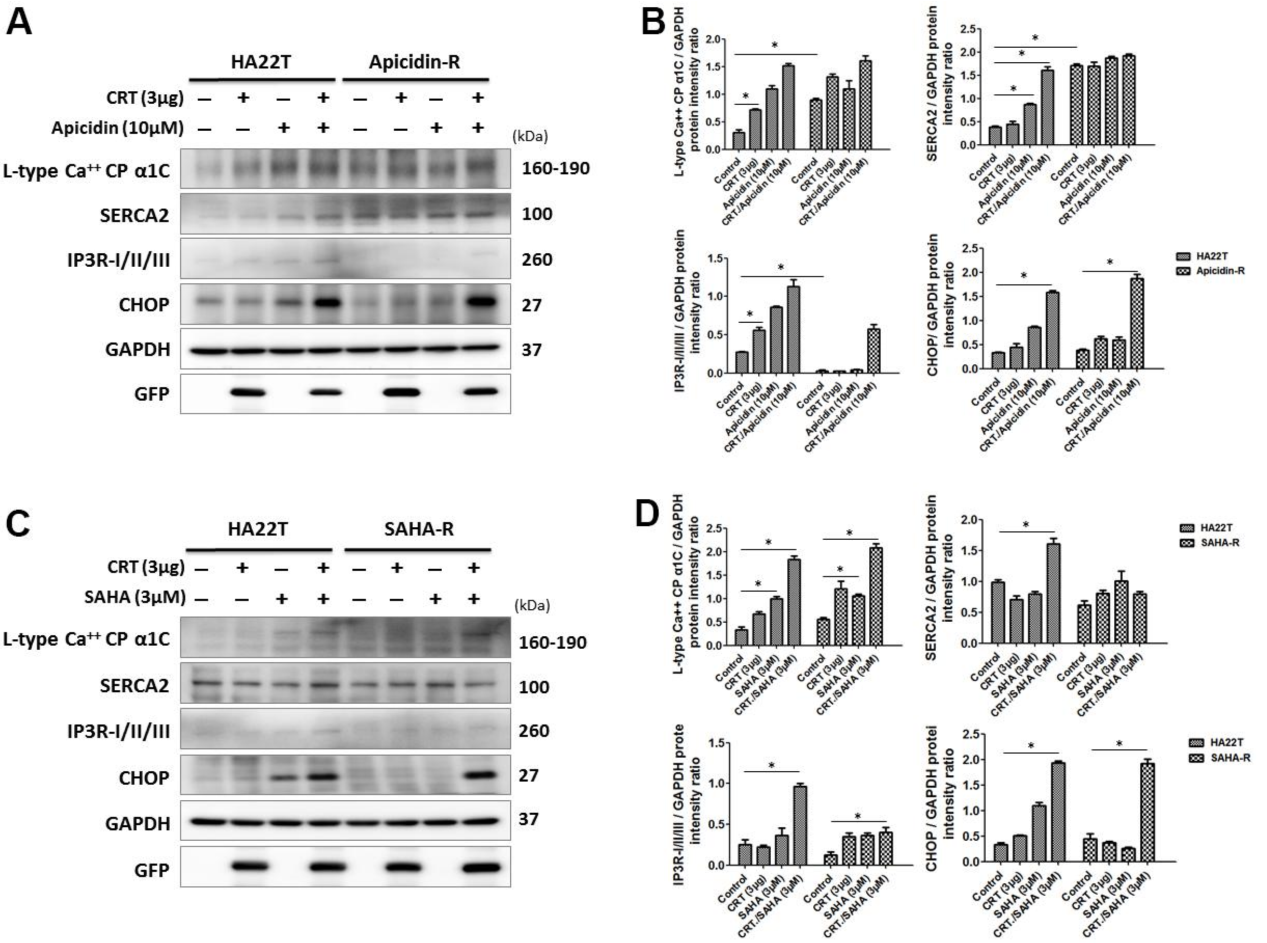

Supplementary Figure 3. The expression of Ca channel-related markers in HCC cells. (**A**–**D**) HCC cells treated with vehicle, transfected with CRT-plasmid, or HDAC inhibitors alone or in combination. The expression of apoptotic proteins is assessed by western blotting. All protein samples are analyzed by western blotting. Protein expression is normalized to expression of GAPDH. *p<0.05, **p<0.01, ***p<0.001 compared with control group.
